# Exploring Bovine Serum Albumin (BSA) as a Facile Substrate for Testing of Osmolytes as Cosmetic Ingredients

**DOI:** 10.3390/molecules30030664

**Published:** 2025-02-03

**Authors:** Giulia Sinesi, Lucia Salvioni, Elisabetta Ronchi, Elena Maria Gabriella Barbuzzi, Davide Prosperi, Miriam Colombo, Marco Davide Giustra

**Affiliations:** 1 NanoBioLab, Department of Biotechnology and Bioscience, University of Milano Bicocca, 20126 Milan, Italy; 2 Research & Innovation Department, Intercos SpA, 20864 Agrate Brianza, Italy; 3 Nanobiotechnologies for Health Center, NANOMIB, University of Milano-Bicocca, 20854 Vedano al Lambro, Italy

**Keywords:** osmolytes, protein stability, cosmetic ingredients, bovine serum albumin

## Abstract

Osmolytes, cellular compounds that maintain osmotic balance and integrity, are gaining attention in cosmetics for their ability to modulate protein folding and stability. Their incorporation into hair and skin care products enhances hydration and strengthens cellular structures. As the cosmetic industry seeks innovative and effective ingredients, osmolytes remain promising candidates for further advancing technologies. This study evaluates the protective effects of four osmolytes—betaine, erythritol, glycine, and isopentyldiol—on bovine serum albumin (BSA), as a substrate, under stressors such as UV irradiation, extreme temperatures, pH changes, surfactants, and oxidative stress. Protein stability was assessed via fluorescence emission. Only betaine at 0.50% *w*/*w* provided significant protection under oxidative stress, while glycine was the most effective osmolyte under basic and acidic pH and UVC radiation. Betaine showed potential in mitigating cold-induced stress but increased stress under UVA radiation, highlighting its condition-specific effects. The study emphasizes the need for comprehensive screening and synergistic testing of osmolytes to optimize cosmetic formulations for diverse stressors. Among the tested osmolytes, glycine is a promising candidate for skin and hair care products, though further research is needed. These findings provide a rapid, cost-effective method to evaluate osmolytes’ potential, offering valuable insights for cosmetic formulation.

## 1. Introduction

**Figure 1:**
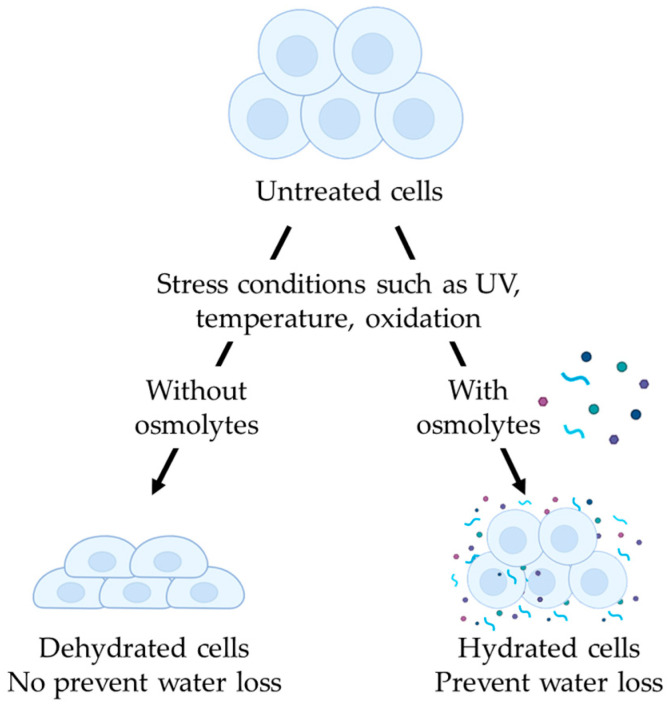
Cell regulation of water loss under stress conditions in the presence or not of osmolytes. Osmolytes guarantee a high level of cell hydration. Created by Biorender.com (accessed on 14 October 2024).

Osmolytes are small organic molecules that belong to various chemical classes, such as methylamines, sugars, amino acids, and polyols. They regulate cellular volume under water stress conditions, including extreme temperatures or UV exposure (Figure 1). Osmolytes stabilize protein structures, modulate aggregation, and influence the solubility of intracellular proteins through the osmophobic effect [1]. Consequently, they help prevent protein denaturation and assist in appropriate protein folding.

Osmolytes can be classified into two categories: protecting osmolytes, which favor the native state (N), and denaturing osmolytes, like urea, which promote the unfolded state (U). Several theories have been proposed to explain their mechanism of action [2,3]. One theory relies on a different interaction with the protein backbone: notably, protecting osmolytes are excluded from the protein surface, increasing water–protein interactions, whereas denaturing osmolytes accumulate at the protein surface. In the case of protecting osmolytes, the interactions between water and protein are stronger, but as the protein unfolds, it exposes more hydrophobic groups, causing repulsive forces to dominate. This result causes an increment in free energy and disfavors the U state due to the polarity of water. Conversely, denaturing osmolytes, which have a higher affinity for the protein backbone, promote the unfolded form [4]. Another explanation for their behavior involves the volume exclusion effect. Since osmolytes have a larger radius than water, they are more easily excluded from the protein vicinity, causing an increase in entropy. This is unfavorable and leads to protein compaction [5].

Thermodynamically, protecting osmolytes shift the equilibrium from the U toward the N state [2,3].

Proteins can interact with each other or their environment through covalent and non-covalent forces. Covalent interactions, such as disulfide bonds, involve the sharing of electrons, while non-covalent interactions include ionic bonds, hydrogen bonds, hydrophobic forces, and electrostatic interactions. When a protein transitions from its unfolded state to its native three-dimensional conformation, no chemical reactions occur; that is, covalent bonds are neither formed nor broken. Instead, this folding process is driven primarily by non-covalent interactions. Electrostatic interactions, hydrophobic effects, steric hindrance, hydrogen bonds, and Van der Waals forces are crucial for maintaining the structural integrity of proteins under stress conditions [6,7,8,9,10]. Osmolytes influence these weak forces by modifying the protein’s surrounding aqueous environment, which can stabilize or destabilize the protein structure depending on the type of osmolyte and the specific stressor. The protective or destabilizing effects of osmolytes on proteins under various stress conditions are directly linked to their ability to modulate these non-covalent interactions, highlighting their importance in protein stability mechanisms.

Given that human hair and skin are often subjected to stress factors such as bleaching, dehydration, and UV exposure, osmolytes are extensively utilized as ingredients in cosmetic formulations due to their ability to protect and stabilize proteins, cells, and tissues under stress conditions [2,3,4]. These protective osmolytes help safeguard skin and hair by minimizing cellular damage from environmental stressors such as extreme temperatures, UV radiation, and pollution. In hair care, they play a vital role in protecting keratin, preventing damage from processes like bleaching or dyeing, and improving moisture retention in the hair shaft. Their multifunctionality positions osmolytes as key components in formulations aimed at promoting skin and hair health.

**Figure 2:**
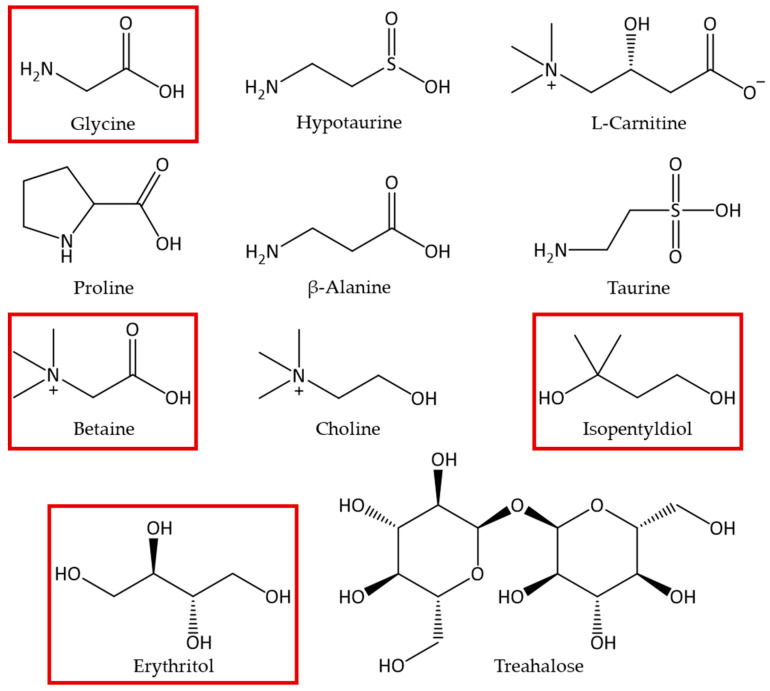
List of some osmolytes used in cosmetic formulations, and four of them were selected (red squares) for investigating their protection property under stress conditions.

In this study, we selected four protecting osmolytes commonly used in cosmetic formulations (i.e., betaine, isopentyldiol, erythritol, and glycine (Figure 2)) to compare their osmolytic properties to benefit final customers.

Betaine, an especially effective osmoprotectant, is a zwitterionic compound and can accumulate inside cells when exposed to osmotic shock, assisting in cell volume regulation [11,12]. In cosmetics, betaine is commonly used as an antioxidant and anti-inflammatory ingredient and is present in many products: hair sprays, body and hand products, non-coloring hair powders, and indoor tanning preparations. For instance, betaine concentration in hair sprays is up to 3%, and in non-coloring hair powders up to 0.0001% [13].

Similarly, polyols such as erythritol and isopentyl diol accumulate in cells under stress conditions, stabilizing globular proteins through hydrogen bonding [14,15]. In hair care and skin care, erythritol is typically used in the 3–5% range. Its function in cosmetics and personal care products is moisturizing. Isopentyldiol is used in face powders at concentrations up to 0.33%, and in perfumes and aerosol deodorants at concentrations up to 5% and up to 1%, respectively [16]. It is also reportedly used as a preservative in personal skin care products between 1.0% and 5.0% by weight [17].

Glycine, the simplest proteinogenic amino acid, functions as an osmoprotectant by accumulating intracellularly under hyperosmotic stress, balancing osmotic pressure and preventing cellular dehydration [18]. Its zwitterionic nature allows it to interact with both positively and negatively charged groups [19], which contributes to protein stabilization by enhancing protein solubility and preventing denaturation. Moreover, glycine acts by stabilizing macromolecular structures through electrostatic and hydrogen bonding interactions, supporting cellular integrity under extreme osmotic conditions. This mechanism helps preserve the structure and function of proteins and other macromolecules during stress. Amino acids like glycine help protect proteins by counteracting saline stress. In cosmetic formulations, glycine is primarily used as a hair and skin conditioning agent, a common benefit shared by many amino acids. Additionally, it serves as a buffering agent and pH adjuster. In general, glycine is reportedly used in a concentration range of 0.0005% to 4%. It is used in a concentration of 2% in moisturizers, 1% in shave and cuticle creams, 0.025% in a face and neck product, and up to 0.3% in a face spray [20].

In this study, we evaluated how osmolytes can moderate the stress that proteins undergo in extreme conditions. Albumins, which regulate oncotic pressure, are commonly used in pharmaceutical and biomedical applications due to their low toxicity, high solubility, biodegradability, and low cost [21]. Specifically, bovine serum albumin (BSA) was selected as a substrate for this study. BSA shares 76% structural homology with human serum albumin (HSA), making it a suitable choice for our investigation. BSA was selected for comparison due to its complex structure, disulfide bonds, and amino acid composition, which are similar to those of HSA, making it a suitable substrate protein for studying structural stability, binding interactions, and the effects of various environmental conditions on protein behavior. The thermal denaturation of BSA has been extensively studied, with particular focus on the stabilizing effects of osmolytes like glycine and betaine under stress conditions [6,19]. Additionally, research has examined the effects of polyols on proteins like BSA, emphasizing their role in preferential hydration [22,23]. However, specific studies on erythritol and isopentyldiol are lacking. Beyond thermal stability, this research also explores the impact of pH, solution components, UV light, and bleaching treatments on BSA. This comprehensive analysis aims to enhance the understanding of BSA’s behavior under various environmental conditions, which is critical for its applications.

Our study provides a novel perspective on the use of osmolytes in cosmetic applications, emphasizing their role in enhancing protein stability and, subsequently, improving formulation performance. We investigated concentrations relevant to cosmetic formulations, taking into account the need to balance osmolyte levels before the formulation phase to avoid undesirable effects, such as changes in texture (e.g., scrub-like sensations) or compromised emulsion stability in products like shampoos and creams.

A systematic evaluation of four osmolytes—betaine, erythritol, glycine, and isopentyldiol—was conducted under common stress conditions, highlighting the specific efficacy of each compound. Considering that the literature on certain osmolytes, such as glycine, is both limited and outdated, our study fills a critical gap by demonstrating glycine’s significant potential to mitigate protein stress under various conditions. The methodology used, rapid and cost-effective, provides a solid foundation for the development of advanced formulations and serves as a useful tool for preliminary screening of molecules for cosmetic products. The obtained results suggest new opportunities to optimize the selection of ingredients based on environmental and biological stress conditions.

## 2. Results

### 2.1. Rationale for Setting the Testing Range Concentration

In cosmetics, the concentration of osmolytes in a formulation is important for several reasons, including how well it moisturizes, how well the active ingredients work, and how the product feels and stays stable. Humectant osmolytes, such as glycerol and sorbitol, act as essential stabilizers in preventing phase separation in emulsions, promoting cohesion between the aqueous and lipid components of the formulation. The presence of osmolytes is often relevant in products susceptible to imbalances caused by thermal or mechanical variations. However, determining the optimal concentration of osmolytes in a formulation is a critical step in the development of cosmetic products. An insufficient amount may not provide adequate protection against dehydration, while an excessive concentration could negatively affect the sensory and rheological properties of the final product. An osmolyte excess may create a sticky feel or alter the viscosity, impacting the sensory effect. Hence, in all the experiments we performed we set a concentration range below 2% before performing the stress treatments to simulate the osmolyte content typically found in a cosmetic product. As noted, the osmolyte concentration is relatively low and varies depending on the formulation.

### 2.2. Evaluation of Protein Stability Under Stress Conditions

BSA was selected as a substrate to evaluate its stability in the presence of osmolyte under common stress factors in beauty treatments. The protective effect of the osmolyte towards denaturation has been studied with spectrofluorometric analyses by investigating the tertiary structure. The protein concentration was set at 0.5 mg/mL in PBS 10 mM pH 7.2 as standard by measuring emission fluorescence at 340 nm of amino acid residues such as tryptophan (Trp), tyrosine (Tyr), and phenylalanine (Phe) [24] exciting at 280 nm. The literature shows tertiary protein folding evaluations that consider the emission intensity. More specifically, a decreased fluorescence intensity is related to a conformational protein change provoked by the unfolding or the denaturation [25,26,27,28]. Instead, a shift in the maximum emission indicates a change in the chemical environment, driven by interactions between the protein and the solvent, surfactant, or ions. This makes it especially intriguing to monitor the effect based on the specific test conducted.

Experiments were conducted to mimic stress conditions in which hairs and skin are usually involved. Thus, the following tests were chosen:

- Oxidation using 20 volumes of H_2_O_2_ (~6.00% *w*/*v*) to simulate a condition for hair dyeing, even if common commercial bleaching agents contain also persulfate salts [29]. The chosen concentration allows for lightening the hair by 1 to 2 levels of lift (results vary depending on the natural hair color) and is especially used by individuals with difficult-to-cover gray hair. Usually, this concentration is combined with bleach to enhance the powerlifting. Treatments using 30 (~9.00% *w*/*v*) or 40-volume (~12.00% *w*/*v*) developers are more extreme and suitable for bleaching thicker hair and achieving lighter colors [30,31]. Additionally, hair bleaching treatments are known to be extremely aggressive, damaging the cuticles and leaving hair weak and dull. Understanding whether a specific osmolyte can mitigate this damage under such stress conditions is crucial for developing a formulation that protects hair and prevents damage to the scalp.

**Figure 3:**
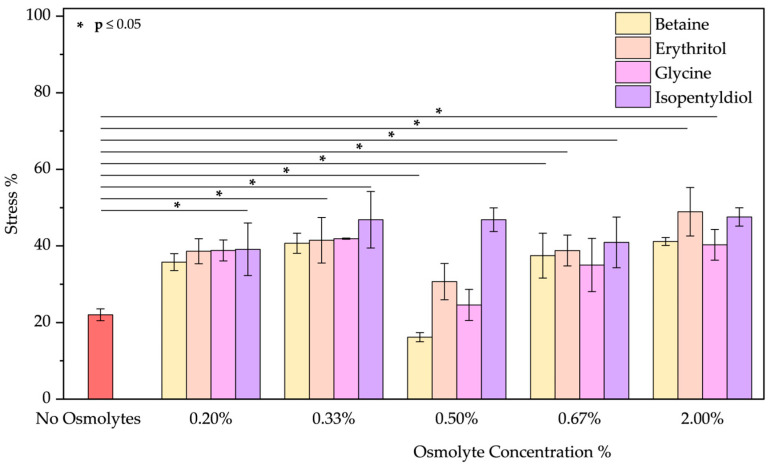
Stress percentage comparison of BSA in the presence or not of osmolyte at 0.20, 0.33, 0.50, 0.67, and 2.00% *w*/*w* by applying oxidative treatment. Data represent mean ± SD (n = 3). *p*-value, * *p* < 0.05, compared to the control (no osmolytes), as indicated by horizontal lines with asterisks. The absence of a line indicates no significant difference.

After oxidative treatment, BSA was analyzed by measuring its fluorescence emission. In the Supplementary Information, Figure S1a–d illustrates a decrease in peak intensity, indicating conformational changes in the protein structure. Specifically, we compared the applied stress based on the relative osmolyte concentrations. Figure 3 shows the percentage of stress of BSA in the presence or absence of the selected osmolytes. Our observations indicate that co-incubation with osmolytes significantly increases the stress levels, in some cases nearly doubling the value to around 45%. Only in one specific case, with high reproducibility, we found that betaine at 0.50% *w*/*w* enhances the protein stability. The non-concentration dependency of betaine is notable, given that it seems to affect stability only at a specific concentration. This unwanted effect is likely due to the interaction of these molecules with H_2_O_2_.

Notably, erythritol could be directly oxidized, leading to the formation of erythrose (Figure S14) [32], whereas, glycine may participate in hydrogen bonding interactions with H₂O₂, potentially altering the reactivity and stability of the oxidant [33,34].

- Thermal stress was applied using working temperatures of 70 °C and 4 °C. The first condition was selected to simulate hair drying, even though the transition in the amorphous phase of keratin occurs at 155 °C and the melting/denaturation of the α-crystalline phase at 233 °C. The temperature of 70 °C is significant because it marks the point at which loosely bound water is removed from the hair [35]. Additionally, the denaturation of BSA occurs at 65 °C making this protein a suitable substrate for studying stress-induced changes in protein conformation [36].

Furthermore, cold temperatures (4 °C) were tested to emulate typical winter conditions or cold-water rinses, which are known to close the hair cuticles, resulting in increased shine. Exposure to low temperatures is also relevant in skincare and beauty treatments.

**Figure 4:**
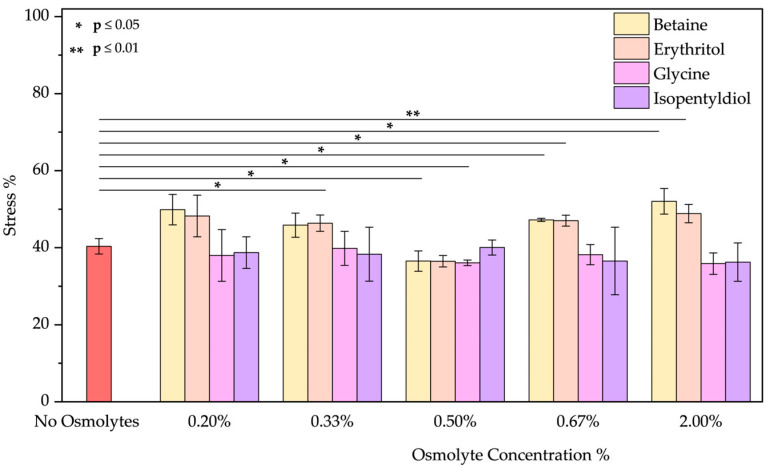
Stress percentage comparison of BSA in the presence or not of osmolyte at 0.20, 0.33, 0.50, 0.67, and 2.00% *w*/*w* by applying high temperature (70 °C). Data represent mean ± SD (n = 3). *p*-value, * *p* < 0.05 and ** *p* < 0.01, compared to the control (no osmolytes), as indicated by horizontal lines with asterisks. The absence of a line indicates no significant difference.

❖ High-Temperature Stress: The thermal stress at 70 °C influences BSA negatively. Figure S2a–d shows that the protein had a significant conformational change, evidenced by a blue shift of about 10 nm, indicating that tryptophan resides in a more hydrophobic environment. The osmolytes did not have a significant protective effect on the protein structure, except for glycine (which caused a red shift of 5 nm), as shown by the spectra, which were similar to the treated control (referred to as ‘no osmolyte’, Figure S2a–d) [37,38]. Comparing the high-temperature stress effect on BSA with and without osmolytes (Figure 4), betaine and erythritol between 0.20 and 2.00% *w*/*w* seem to enhance this stress resulting in a worsened condition on tertiary structure.

**Figure 5:**
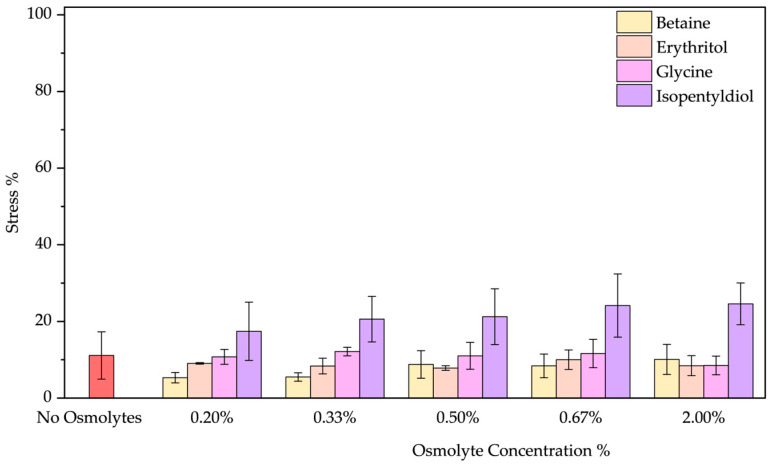
Stress percentage comparison of BSA in the presence or not of osmolyte at 0.20, 0.33, 0.50, 0.67, and 2.00% *w*/*w* by applying low temperature (4 °C). Data represent mean ± SD (n = 3). *p*-value compared to the control (no osmolytes), no significant difference is observed (absence of horizontal lines with asterisks).

❖ Low-Temperature Stress: The thermal stress at 4 °C influences BSA negligibly showing a stress below 10%. However, only by adding betaine below 0.33%, the stress effect drops to 5–6%, by increasing the concentration no effect was noted (Figure 5). The presence of glycine and erythritol does not affect the stability as Figure S3a–d shows, but isopentyldiol allows a blue shift of 1–2 nm (increasing with concentration). In fact, isopentyldiol plays a negative role in increasing protein instability (stress 18–25%). Figure 5 shows better the effect of osmolytes by treating BSA at 4 °C. Among the data obtained, betaine addition appears beneficial at low concentrations (below 0.33% *w*/*w*).

**Figure 6:**
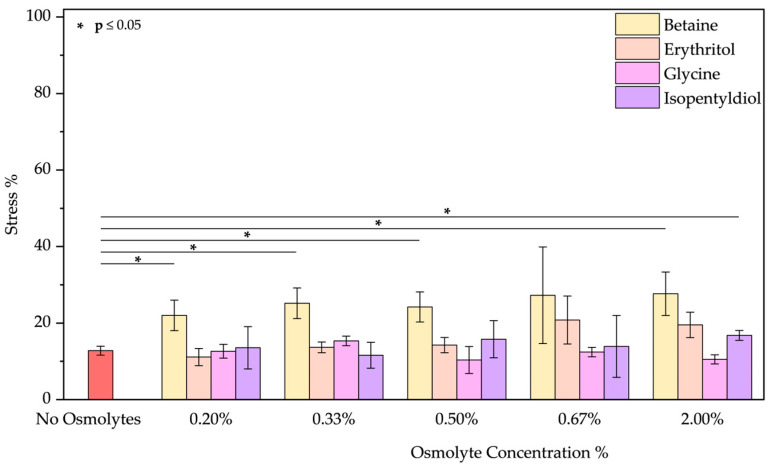
Stress percentage comparison of BSA in the presence or not of osmolyte at 0.20, 0.33, 0.50, 0.67, and 2.00% *w*/*w* by applying UVA radiation. Data represent mean ± SD (n = 3). *p*-value, * *p* < 0.05, compared to the control (no osmolytes), as indicated by horizontal lines with asterisks. The absence of a line indicates no significant difference.

- UV irradiation was applied using a UV lamp to simulate skin or hair exposure to sunlight. Ultraviolet (UV) rays are classified into three main categories based on wavelength: UVA (320–400 nm), UVB (280–320 nm), and UVC (100–280 nm) [39]. UVC rays do not reach the Earth’s surface due to absorption by the ozone layer. However, following the pandemic, the improper use of UVC lamps as germicidal agents for surface and hand sterilization, combined with the reduction in the ozone layer, has made this type of radiation a potential risk factor for the skin and eyes [40]. UVA and UVB rays remain the primary sources of exposure for humans and are responsible for both short-term effects, such as erythema and sunburn, and long-term effects, including photoaging and an increased risk of skin cancers. Although UVB rays are essential, in low doses, for vitamin D production, prolonged exposure to UV rays can also cause significant hair damage [39]. Specifically, UVA irradiation can degrade the cuticles, reducing moisture content and making hair dry, brittle, and frizzy, especially in individuals with curly or naturally dry hair. Also, melanin can be degraded, increasing hair discoloration and a dull appearance. The emission spectrum (Figure S4a–d) after UVA treatment shows a small reduction in intensity, suggesting a slight change in the environment around tryptophan and tyrosine residues, particularly in the presence of betaine [41,42]. The other osmolytes had similar effects, with the best results achieved by glycine above 0.50% *w*/*w* (Figure 6).

We conducted experiments on BSA using UVC radiation, as shown in Figure S5a–e. Although UVC rays do not significantly affect the BSA structure, betaine was able to protect the protein, reducing stress to 0%. Additionally, glycine provided protection, effectively reducing stress at concentrations below 0.67% *w*/*w*. Betaine and glycine behave differently, with betaine modulating the effects of UV wavelengths and glycine offering direct UV protection.

- pH stress was applied using a basic solution at pH 12 and an acid solution at pH 2 to simulate significant damage to the hair structure caused by applying harsh products. The pH level of hair products significantly affects the hair shaft. At an alkaline pH, hair’s water sorption increases, and hydrolysis temporarily breaks hydrogen bonds, making keratin more malleable and weakening the hair shaft. At pH levels above 10, hydroxyl group sorption rises, breaking salt bridges and disulfide bonds and hence damaging the hair structure [43,44]. Alkaline products, such as high-pH shampoos, increase the negative charge on fiber surfaces, leading to greater friction between fibers, breaking them, and damaging cuticles [45]. Conversely, at pH levels below 4, hydrogen ion sorption increases significantly. Carboxylic groups are protonated, substituting saline bridges with hydrogen bonds, further weakening the hair shaft. Peptide bonds may undergo hydrolysis below pH 2, causing irreversible changes [44]. Indeed, hair straightening products with pH values below 2 can cause considerable damage to the hair [46]. Moreover, the skin can also be affected by the pH of products that disturb this barrier, potentially leading to increased pH, weakened skin cohesion, dryness, irritation, and altered microflora [47].

**Figure 7:**
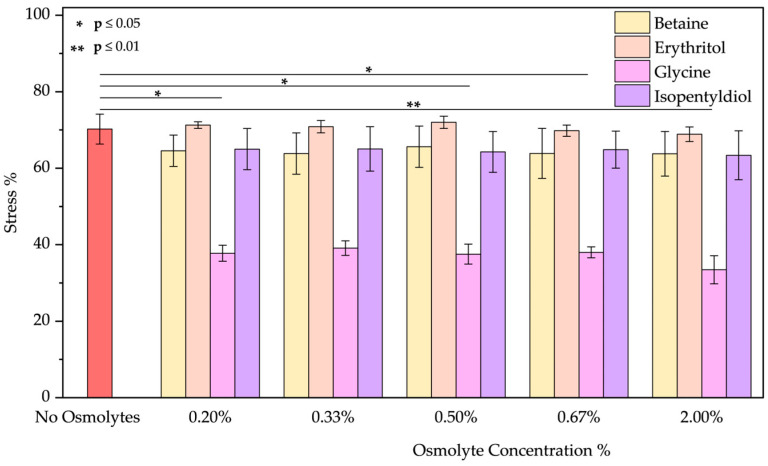
Stress percentage comparison of BSA in the presence or not of osmolyte at 0.20, 0.33, 0.50, 0.67, and 2.00% *w*/*w* by applying basic pH. Data represent mean ± SD (n = 3). *p*-value, * *p* < 0.05 and ** *p* ≤ 0.01, compared to the control (no osmolytes), as indicated by horizontal lines with asterisks. The absence of a line indicates no significant difference.

❖ Basic pH: As the emission spectrum shows, BSA adopts an “aged form” losing its tertiary structure at pH 12. The emission peak decreases due to the unfolding of the protein, as expected [48,49,50]. A blue shift of 12 nm suggests tryptophan is in a less polar environment. Only glycine plays a role in the unfolding of BSA. Upon detailed examination of all the spectra, it is evident that, except for glycine, the other osmolytes induced a blue shift (Figure S6a–d). Glycine showed the best results in reducing the stress % from 70.23 ± 3.90% to 33.45 ± 3.68% (value obtained using glycine at 2.00% *w*/*w*; applying lower concentrations the results are similar as Figure 7 demonstrates).

**Figure 8:**
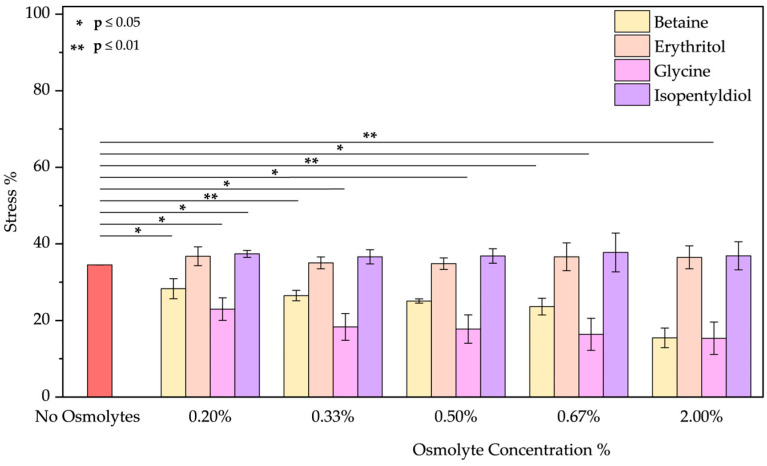
Stress percentage comparison of BSA in the presence or not of osmolyte at 0.20, 0.33, 0.50, 0.67, and 2.00% *w*/*w* by applying acid pH. Data represent mean ± SD (n = 3). *p*-value, * *p* < 0.05 and ** *p* ≤ 0.01, compared to the control (no osmolytes), as indicated by horizontal lines with asterisks. The absence of a line indicates no significant difference.

❖ Acid pH: As the emission spectrum shows, BSA assumes an “expanded form” due to a decrease in its negative charge at pH 2. Due to the exposure of hydrophobic groups, a blue shift of 10 nm was observed. Osmolytes slightly mitigated protein unfolding (Figure S7a–d) where a red shift was noted by increasing the glycine concentration (passing from 328 to 335 nm). Betaine and glycine show the most significant effects at 2.00% *w*/*w* reducing acid stress from 34.52 ± 0.01% to 15.45 ± 2.57% and 15.36 ± 4.23%, respectively (Figure 8). Even if, we tested extreme acid pH, these two molecules can be considered to diminish this stress.

**Figure 9:**
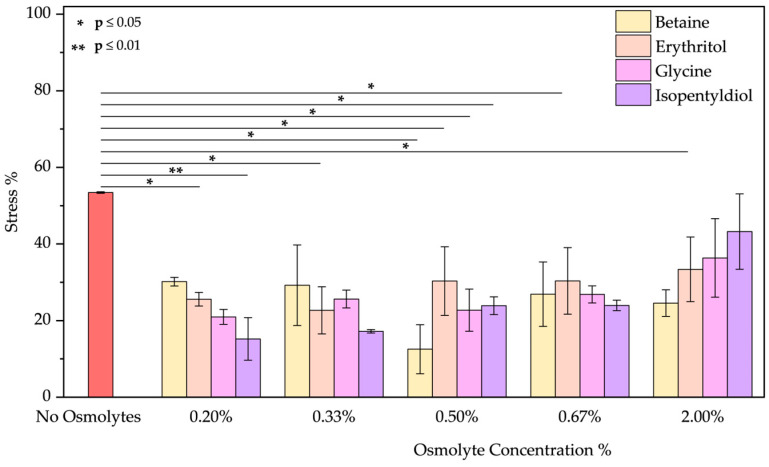
Stress percentage comparison of BSA in the presence or not of osmolyte at 0.20, 0.33, 0.50, 0.67, and 2.00% *w*/*w* by applying acid peeling solution as stress effect simultaneously. Data represent mean ± SD (n = 3). *p*-value, * *p* < 0.05 and ** *p* ≤ 0.01, compared to the control (no osmolytes), as indicated by horizontal lines with asterisks. The absence of a line indicates no significant difference.

**Figure 10:**
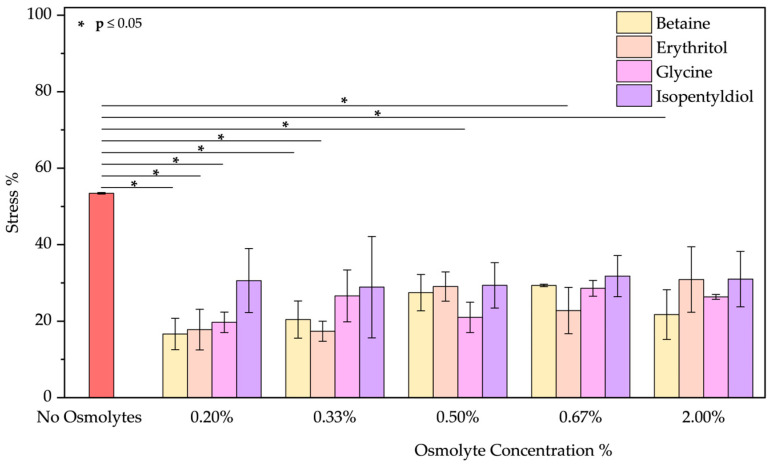
Stress percentage comparison of BSA in the presence or not of osmolyte at 0.20, 0.33, 0.50, 0.67, and 2.00% *w*/*w* by applying acid peeling solution as stress effect after treatment of osmolytes. Data represent mean ± SD (n = 3). *p*-value, * *p* < 0.05, compared to the control (no osmolytes), as indicated by horizontal lines with asterisks. The absence of a line indicates no significant difference.

**Figure 11:**
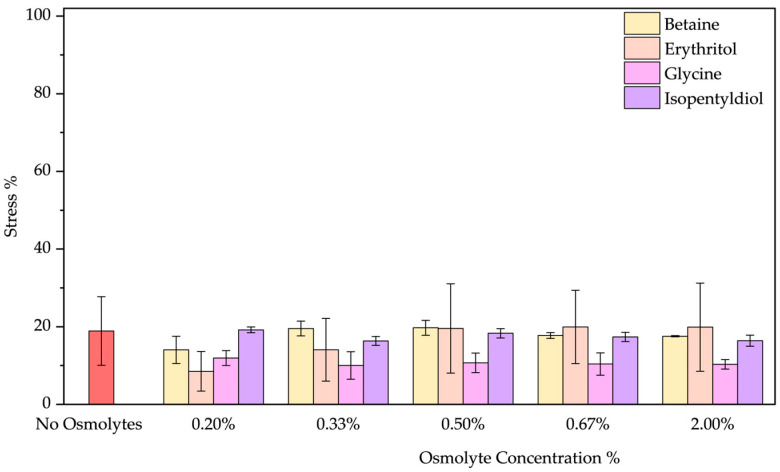
Stress percentage comparison of BSA in the presence or not of osmolyte at 0.20, 0.33, 0.50, 0.67, and 2.00% *w*/*w* by applying SLES solution. Data represent mean ± SD (n = 3). *p*-value compared to the control (no osmolytes); no significant difference is observed (absence of horizontal lines with asterisks).

- The peeling treatment was simulated using an acid solution at pH 3.5, composed of typical molecules commonly found in skin acid peeling products. A chemical exfoliating agent is applied to stimulate rejuvenation, smoothness, and improvement of the skin’s superficial structure through controlled chemical damage. For instance, α-Hydroxy acids (AHAs), such as glycolic and lactic acid, are mild acids commonly used in these procedures. They promote exfoliation at low concentrations (<30%) by reducing sulfate and phosphate groups. However, at higher concentrations, they cause more significant damage to the epidermis [51,52]. To simulate the stress induced by these treatments, we performed two distinct protocols. In the first trial, BSA was treated with a solution of glycolic (5.70%), lactic (2.15%), and citric acid (2.15%) at pH 3.5 in the presence of osmolyte. In the second one, BSA was treated with this acid solution after the incubation with an osmolyte. These two approaches were designed to emulate common skincare routines: one where the protective component is included in the peeling solution and another where the peeling treatment is applied after a protective product. In both trials, the acid peeling treatment caused a blue shift of 10 nm and a decrease in peak intensity resulting in a stress of 53.43 ± 0.19%. However, osmolytes in both procedures provided significant benefits (Figures S7a–d and S8a–d). In the first treatment, where the protein is in the “fast form” [53] (similar situation in acid pH stress), isopentyldiol offered greater protection at lower concentrations. The other three osmolytes keep stress levels below 30% (Figure 9). As noted previously (Figure 3, in the Oxidative stress and Figure 4 in the High-Temperature Stress), betaine exhibits unusual behavior, showing a non-linear concentration dependence (V-shaped trend), with greater benefits observed at an intermediate concentration among those tested. In the second protocol, except for isopentyldiol, which shows benefits, lower osmolyte concentrations generally result in stress percentages ranging between 16% and 19% (Figure 10). It is crucial to provide the appropriate procedures to enhance the efficacy and prevent potential permanent damage. Overall, all four osmolytes demonstrated a positive effect, making them highly recommended for further in vitro testing.

- Surfactant treatment was performed by incubating BSA in a sodium lauryl ether sulfate (SLES) solution at 0.50 mg/mL. Surfactants are found in many cosmetic products and cleaning agents due to their ability to reduce interfacial tension between phases. In shampoos and conditioners, they can partially remove hair lipids, which exposes hair fibers to greater damage [54]. Since lipids are essential for protecting hair from environmental and chemical damage, surfactants may also interact negatively with epidermal proteins or lipids, leading to skin irritation [55]. SLES was chosen as a representative surfactant for this study. In Figure S10a–d, the emission spectrum shows a blue shift of 17 nm, indicating a more hydrophobic environment due to the application of SLES. None of the osmolytes significantly promote re-obtain initial protein conformation and not exposing the fluorescent residues (tryptophan or tyrosine) from the aqueous surroundings [56,57,58]. Among the osmolytes (Figure 11), betaine and erythritol seem to reduce stress when applied at a concentration of 0.20% (14.04 ± 3.49% and 8.52 ± 5.09%, respectively). Glycine displayed better results overall in terms of the range of applicable concentrations as showed reducing from stress of 18.89 ± 8.84% to values around 10–12%.

## 3. Discussion

In this study, we examined the effects of nine treatments on a protein, BSA, in the presence of selected osmolytes. Our analysis aimed to determine whether these treatments altered the conformational structure of this protein (Figures S12 and S13) and how osmolytes influenced these changes under different stress conditions. This investigation was conducted using an inexpensive protein that serves as a useful tool for chemists and formulators to screen molecules before their incorporation into a potential product. The results indicate that the effects of osmolytes, except in rare cases, do not significantly depend on their concentration.

The osmolyte concentrations were maintained below 2% to avoid too much solute per water and have the opposite effect, including dehydration and denaturation. The data obtained from the stress exposure to BSA are summarized in Table 1.

In summary, we can state that under oxidative stress, only adding betaine at 0.50% *w*/*w* provided protection. The other osmolytes significantly affected the protein stability, indicating that they are not suitable candidates as ingredients during bleaching treatment. At 70 °C, the selected osmolytes were not able to effectively protect the protein. Our results suggested that these molecules are not recommended unless the synergistic action of a mixture of osmolytes could provide significant benefits. On the other hand, after the treatment at 4 °C, isopentyldiol addiction negatively impacts protein stability, whereas the low concentration of betaine appears to reduce cold-induced stress. The latter could be beneficial in preventing dehydration phenomena during winter in formulations such as lip balms. Under UVA irradiation, the protein did not exhibit any conformational changes. However, the use of betaine increased radiation-induced stress, as did erythritol and isopentyldiol. Under UVC radiation, betaine effectively shielded and protected the protein, as did glycine. Exposing BSA to basic pH, only glycine demonstrated the ability to protect the protein, reducing the stress by more than 50%. The other osmolytes did not appear to provide significant benefits. At acidic pH, betaine and glycine significantly enhance protein stability in a concentration-dependent manner. It is worth noting that, unlike the previous cases, the extent of the effect ranges from moderate to good based on our scale. Hence, it is advisable to include concentrations around 2% or higher in the final product.

When simulating the acid peeling treatment, a significant effect was observed with all the osmolytes both in co-incubation and pre-incubation settings. Notably, the strength of this protection varies depending on the molecule and the addition time. These results are particularly interesting for determining the optimal application method of a potential product in skincare routines to maximize benefits. Finally, a moderate impact was noted also when incubating BSA with SLES solution in the presence of glycine and low concentrations of betaine and erythritol.

The overall results of this study confirm that it is highly desirable to perform a comprehensive screening of all molecules intended for use in a formulation for a specific treatment. Additionally, testing mixtures of molecules is challenging due to the wide variety of components found in cosmetic formulations. For a company and its research and development department, having a database capable of cross-referencing various experimental outcomes is not trivial, but it is crucial for enhancing the effectiveness of a cosmetic product. This is especially important with the advent of artificial intelligence [59,60], which can assist formulation chemists by providing insights, considering that in daily life, we are often subjected to multiple stressors simultaneously, such as exposure to UV radiation, high temperatures, and the possible presence of SLES in cosmetic products (synergistic stress effects).

It is also crucial to differentiate between types of stress, regardless of whether they are classified as high or low because even a 10% stress level can be extremely significant in the context of skin and hair. For companies, it is essential to develop experimental models that are cost-effective yet highly reliable during the formulation phase to avoid misleading advertising and ensure the safety and effectiveness of their products.

In any case, although their effects could not be considered of general applicability, osmolytes exhibited a protective capacity against BSA denaturation in several cases, as observed in the spectral analysis. Glycine consistently showed the best protective effect, likely due to its specific mechanism of action. Being a zwitterion, glycine can interact with protein through its charged groups facilitated by its little dimension. It is suggested that below 100 mM of amino acid concentration, the stability of the protein is due to the interactions between the glycine and the peptidic structure [19]. As a result of BSA denaturation, the protein surface area is expected to increase, thus exposing more hydrophobic groups to the solvent. In the first case, the denatured state has higher energy for the increment of the transfer-free energy due to the volume exclusion of glycine. In the second one, the transfer-free energy of the unfolded protein is larger due to the repulsion forces between the hydrophobic groups and water, stabilizing the native state [6].

In conclusion, this study highlights the potential of glycine as an effective osmolyte in protecting protein structures, while further research is needed to fully disclose its behavior across different conditions. Moreover, to set a study useful for researchers especially in research and development to obtain the best product possible for skin and hair protection.

More studies will be conducted to translate what was observed in the various treatments in various cosmetic formulas, exploiting conventional models, e.g., mammalian skin and virgin hairs.

## 4. Materials and Methods

### 4.1. Materials and Chemicals

Betaine, bovine serum albumin (BSA), citric acid monohydrate, erythritol, glycine, hydrochloric acid (HCl), glycolic acid, hydrogen peroxide 30% *w*/*w* in water (H_2_O_2_), isopentyldiol, lactic acid, sodium chloride (NaCl), sodium ethyl laurate sulfate (SLES), sodium hydroxide (NaOH), sodium phosphate dibasic, and monobasic were purchased from Sigma Aldrich. Water used in all protocols was purified by passing through the MilliQ Millipore system.

### 4.2. Evaluation of Protein Stability Under Stress Conditions

For the stress treatments, 0.5 mg/mL of bovine serum albumin (BSA) was prepared in phosphate-buffered saline (PBS) at 10 mM pH 7.2.

After the treatments were performed by following the protocols shown below, the tertiary structure of BSA was analyzed with the JASCO Spectrofluorometer FP8500 using a quartz cuvette. The samples were measured at 83.3 µg/mL. Each spectrum was collected from 290 to 450 nm by exciting at 280 nm, the time response was 1 s, and the scan speed of 200 nm/min.

The data were expressed as stress percentages, normalized to untreated BSA (stress set at 0%) following Equations (1) and (2).

(1) $$\mathrm{Stress}\%=(1-{\mathrm{BSA}}_{\mathrm{yield}})\%$$

(2) $${\mathrm{BSA}}_{\mathrm{yield}}=\frac{{\mathrm{BSA}}_{\mathrm{treated}}}{{\mathrm{BSA}}_{\mathrm{untreated}}}$$

For BSA_treated_ and BSA_untreated_ were considered the relative maximum emission fluorescence peak for each concentration and treatment. Calculations were performed on three independent experiments. Fluorescence emission of osmolytes at the maximum concentration (2.00% *w*/*w*) was acquired to exclude interference (Figure S11).

#### 4.2.1. Oxidative Stress

BSA was dispersed at 0.5 mg/mL in PBS 10 mM pH 7.2 as a physiological buffer in the presence of 20 volumes of H_2_O_2_ (~6.00% *w*/*v*). The protein solutions were incubated in the presence of osmolyte at 0.20%, 0.33%, 0.50%, 0.67%, and 2.00% *w*/*w* as final concentrations stirring for 2 h at 37 °C. The characterization of solutions was performed by exciting at 280 nm.

#### 4.2.2. Thermal Stress

BSA was dispersed at 0.5 mg/mL in PBS 10 mM pH 7.2 in the presence of osmolyte at 0.20%, 0.33%, 0.50%, 0.67%, and 2.00% *w*/*w* as final concentrations stirring for 2 h at 70 °C. The characterization of solutions was performed by exciting at 280 nm. BSA was dispersed at 0.5 mg/mL in PBS 10 mM pH 7.2 in the presence of osmolyte at 0.20%, 0.33%, 0.50%, 0.67%, and 2.00% *w*/*w* as final concentrations stirring for 2 h at 4 °C. The characterization of solutions was performed by exciting at 280 nm.

#### 4.2.3. UV Radiation

BSA was dispersed at 0.5 mg/mL in PBS 10 mM pH 7.2 in the presence of osmolyte at 0.20%, 0.33%, 0.50%, 0.67%, and 2.00% *w*/*w* as final concentrations stirring for 2 h at 37 °C exposing to UV light. The characterization of solutions was performed by exciting at 280 nm.

#### 4.2.4. pH Stress

BSA was dispersed at 0.5 mg/mL in 0.01 M NaOH (pH 12) in the presence of osmolyte at 0.20%, 0.33%, 0.50%, 0.67%, and 2.00% as final concentrations stirring for 2 h at 37 °C. The characterization of solutions was performed by exciting at 280 nm.

BSA was dispersed at 0.5 mg/mL in 0.01 M HCl (pH 2) in the presence of osmolyte at 0.20%, 0.33%, 0.50%, 0.67%, and 2.00% as final concentrations stirring for 2 h at 37 °C. The characterization of solutions was performed by exciting at 280 nm.

#### 4.2.5. Peeling

BSA was dispersed at 0.5 mg/mL in a solution of glycolic (5.70% *w*/*v*), lactic (2.15% *w*/*v*), and citric acids (2.15% *w*/*v*) at pH 3.5 in the presence of osmolyte at 0.20%, 0.33%, 0.50%, 0.67% and 2.00% as final concentrations stirring for 2 h at 37 °C. Two procedures were carried out: first, BSA was incubated in acidic osmolyte solutions; and second, BSA was incubated with osmolytes before acid treatment. The characterization of solutions was performed by exciting at 280 nm.

#### 4.2.6. Surfactant Treatment

BSA was dispersed at 0.5 mg/mL in PBS 10 mM pH 7.2 with SLES (1:1) in the presence of osmolyte at 0.20%, 0.33%, 0.50%, 0.67%, and 2.00% as final concentrations, stirring for 2 h at 37 °C. Solutions were characterized by exciting at 280 nm.

## Figures and Tables

**Table 1:** Stress percentage summary of BSA under stress conditions in the presence or not of betaine, erythritol, glycine, and isopentyldiol at 0.20, 0.30, 0.50, 0.67, and 2.00% *w*/*w* in MilliQ water.

		Stress %								
		Oxidation	70 °C	4 °C	UVA	pH 12	pH 2	Acid Peeling *	Acid Peeling **	SLES
No Osmolyte		22.01 ± 1.54	40.37 ± 2.00	11.13 ± 6.16	12.79 ± 1.16	70.23 ± 3.90	34.52 ± 0.01	53.43 ± 0.20	53.43 ± 0.20	18.89 ± 8.84
Betaine	0.20%	35.78 ± 2.21	49.88 ± 3.95	5.34 ± 1.35	22.01 ± 3.97	64.56 ± 4.10	28.29 ± 2.62	30.16 ± 1.14	16.64 ± 4.09	14.04 ± 3.50
	0.33%	40.71 ± 2.62	45.86 ± 3.14	5.51 ± 1.09	25.18 ± 3.99	63.84 ± 5.40	26.49 ± 1.37	29.23 ± 10.52	20.41 ± 4.86	19.53 ± 1.89
	0.50%	16.20 ± 1.18	36.54 ± 2.65	8.79 ± 3.59	24.20 ± 3.92	65.63 ± 5.39	25.07 ± 0.56	12.54 ± 6.40	27.46 ± 4.76	19.72 ± 1.92
	0.67%	37.48 ± 5.85	47.22 ± 0.40	8.41 ± 3.07	27.27 ± 12.62	63.86 ± 6.54	23.62 ± 2.18	26.89 ± 8.42	29.34 ± 0.32	17.73 ± 0.74
	2.00%	41.17 ± 1.03	52.03 ± 3.32	10.10 ± 3.92	27.67 ± 5.68	63.77 ± 5.83	15.46 ± 2.57	24.56 ± 3.48	21.72 ± 6.49	17.54 ± 0.16
Erythritol	0.20%	38.62 ± 3.25	48.23 ± 5.39	9.05 ± 0.20	11.12 ± 2.22	71.27 ± 0.87	36.79 ± 2.45	25.57 ± 1.78	17.80 ± 5.30	8.52 ± 5.09
	0.33%	41.48 ± 5.94	46.37 ± 2.11	8.36 ± 2.06	13.66 ± 1.41	70.86 ± 1.59	35.06 ± 1.55	22.68 ± 6.14	17.37 ± 2.62	14.07 ± 8.06
	0.50%	30.68 ± 4.75	36.50 ± 1.49	7.85 ± 0.61	14.25 ± 2.00	71.99 ± 1.59	34.85 ± 1.49	30.31 ± 8.97	29.04 ± 3.83	19.56 ± 11.51
	0.67%	38.81 ± 4.03	47.01 ± 1.41	10.01 ± 2.54	20.80 ± 6.25	69.80 ± 1.47	36.65 ± 3.61	30.36 ± 8.69	22.77 ± 6.04	19.93 ± 9.43
	2.00%	48.92 ± 6.32	48.86 ± 2.38	8.46 ± 2.61	19.53 ± 3.33	68.88 ± 1.91	36.50 ± 2.98	33.39 ± 8.43	30.88 ± 8.57	19.89 ± 11.35
Glycine	0.20%	38.84 ± 2.74	38.00 ± 6.70	10.75 ± 1.93	12.65 ± 1.80	37.77 ± 2.09	22.97 ± 2.94	20.95 ± 1.95	19.69 ± 2.67	11.93 ± 1.92
	0.33%	41.89 ± 0.16	39.84 ± 4.41	12.14 ± 1.12	15.34 ± 1.24	39.12 ± 1.91	18.32 ± 3.48	25.63 ± 2.32	26.61 ± 6.79	10.03 ± 3.53
	0.50%	24.59 ± 4.06	36.09 ± 0.72	11.02 ± 3.50	10.36 ± 3.53	37.54 ± 2.62	17.76 ± 3.71	22.71 ± 5.49	20.99 ± 3.97	10.70 ± 2.50
	0.67%	35.01 ± 6.95	38.21 ± 2.62	11.62 ± 3.69	12.43 ± 1.23	37.99 ± 1.43	16.38 ± 4.17	26.82 ± 2.22	28.58 ± 2.07	10.40 ± 2.87
	2.00%	40.29 ± 4.01	35.87 ± 2.78	8.52 ± 2.44	10.53 ± 1.20	33.45 ± 3.68	15.36 ± 4.23	36.36 ± 10.26	26.34 ± 0.62	10.31 ± 1.22
Isopentyldiol	0.20%	39.13 ± 6.86	38.73 ± 4.10	17.42 ± 7.58	13.55 ± 5.52	65.00 ± 5.37	37.41 ± 0.91	15.21 ± 5.56	30.62 ± 8.38	19.20 ± 0.73
	0.33%	46.83 ± 7.38	38.32 ± 6.99	20.59 ± 5.95	11.60 ± 3.38	65.05 ± 5.80	36.64 ± 1.85	17.19 ± 0.45	28.90 ± 13.26	16.33 ± 1.14
	0.50%	46.84 ± 3.08	40.06 ± 1.94	21.23 ± 7.27	15.78 ± 4.85	64.27 ± 5.34	36.86 ± 1.89	23.88 ± 2.32	29.36 ± 5.94	18.30 ± 1.21
	0.67%	40.93 ± 6.62	36.54 ± 8.77	24.15 ± 8.25	13.90 ± 8.07	64.85 ± 4.83	37.78 ± 5.06	23.95 ± 1.37	31.78 ± 5.40	17.37 ± 1.18
	2.00%	47.58 ± 2.40	36.27 ± 4.97	24.57 ± 5.44	16.78 ± 1.29	63.37 ± 6.41	36.89 ± 3.67	43.24 ± 9.84	31.00 ± 7.25	16.40 ± 1.43
	Aggravate					* co-incubation				
	Negligible (variation ≤ 10%)					** pre-incubation				
	Moderate (10% < Variation ≤ 50%)									
	Good (50% < Variation ≤ 75%)									
	Optimal (Variation > 75%)									

## Data Availability

The data used to support the findings of this study are available in the Supplementary Materials and from the corresponding author upon request.

## Supplementary Materials

The following supporting information can be downloaded at: https://www.mdpi.com/article/10.3390/molecules30030664/s1, Figure S1: Evaluation of tertiary structure of BSA exploiting fluorescence emission in the presence or not of osmolyte at 0.20, 0.33, 0.50, 0.67 and 2.00% *w*/*w* by applying bleaching as stress effect; Figure S2: Evaluation of tertiary structure of BSA exploiting fluorescence emission in the presence or not of osmolyte at 0.20, 0.33, 0.50, 0.67 and 2.00% *w*/*w* by applying high-temperature (70 °C) as stress effect; Figure S3: Evaluation of tertiary structure of BSA exploiting fluorescence emission in the presence or not of osmolyte at 0.20, 0.33, 0.50, 0.67 and 2.00% *w*/*w* by applying low-temperature (4 °C) as stress effect; Figure S4: Evaluation of tertiary structure of BSA exploiting fluorescence emission in the presence or not of osmolyte at 0.20, 0.33, 0.50, 0.67 and 2.00% *w*/*w* by applying UVA radiation as stress effect; Figure S5: Evaluation of tertiary structure of BSA exploiting fluorescence emission in the presence or not of osmolyte at 0.20, 0.33, 0.50, 0.67 and 2.00% *w*/*w* by applying UVC radiation as stress effect; Figure S6: Evaluation of tertiary structure of BSA exploiting fluorescence emission in the presence or not of osmolyte at 0.20, 0.33, 0.50, 0.67 and 2.00% *w*/*w* by applying basic solution (pH 12) as stress effect; Figure S7: Evaluation of tertiary structure of BSA exploiting fluorescence emission in the presence or not of osmolyte at 0.20, 0.33, 0.50, 0.67 and 2.00% *w*/*w* by applying acid solution (pH 2) as stress effect; Figure S8: Evaluation of tertiary structure of BSA exploiting fluorescence emission in the presence or not of osmolyte at 0.20, 0.33, 0.50, 0.67 and 2.00% *w*/*w* by applying acid peeling solution as stress effect at the same time; Figure S9: Evaluation of tertiary structure of BSA exploiting fluorescence emission by applying acid peeling solution as stress effect after treatment of osmolyte at 0.20, 0.33, 0.50, 0.67 and 2.00% *w*/*w*; Figure S10: Evaluation of tertiary structure of BSA exploiting fluorescence emission in the presence or not of osmolyte at 0.20, 0.33, 0.50, 0.67 and 2.00% *w*/*w* by applying SLES solution as stress effect; Figure S11: Fluorescence emission of osmolytes at 2.00% *w*/*w*; Figure S12: Absorbance of BSA under stress conditions; Figure S13: FT-ATR of BSA under stress conditions; Figure S14: FT-ATR of Erythritol before and after oxidation treatment.
