# Supplementary material for: Exploring Bovine Serum Albumin (BSA) as a Facile Substrate for Testing of Osmolytes as Cosmetic Ingredients
Source: Molecules. 2025 Feb 3;30(3):664. doi: 10.3390/molecules30030664 (PMC11820442; doi:10.3390/molecules30030664)
Supplement: Supplementary file 1 [file molecules-30-00664-s001.zip › molecules-3374164-supplementary.pdf]

Electronic Supplementary Information

Exploring Bovine Serum Albumin (BSA) as a Facile Substrate for Testing of Osmolytes as Cosmetic Ingredients

Giulia Sinesi [1], Lucia Salvioni [1], Elisabetta Ronchi [2], Elena Maria Gabriella Barbuzzi [2], Davide Prosperi [1,3], Miriam Colombo [1,3,*] and Marco Davide Giustra [1,3,*]

[1] NanoBioLab, Department of Biotechnology and Bioscience, University of Milano Bicocca, 20126 Milan, Italy
[2] Research & Innovation Department, Intercos SpA, 20864 Agrate Brianza, Italy
[3] Nanobiotechnologies for Health Center, NANOMIB, University of Milano-Bicocca, 20854 Vedano al Lambro, Italy
* Correspondence: miriam.colombo@unimib.it (M.C.); marco.giustra@unimib.it (M.D.G.)

1. Evaluation of protein stability under stress conditions exploiting emission fluorescence

Bovine Serum Albumin (BSA) was treated with four osmolytes (Betaine, Erythritol, Glycine, and Isopentyldiol) under stress conditions that skin and hair can be subjected to in daily life. BSA was chosen as a cheap protein substrate to evaluate these molecules, which are used as cosmetic ingredients to protect hair and skin from damage caused by cell dehydration and α-keratin unfolding.
Following exposure to stress conditions, BSA was excited at 280 nm using a spectrofluorometer, and the protein conformation was assessed from the emission spectra.

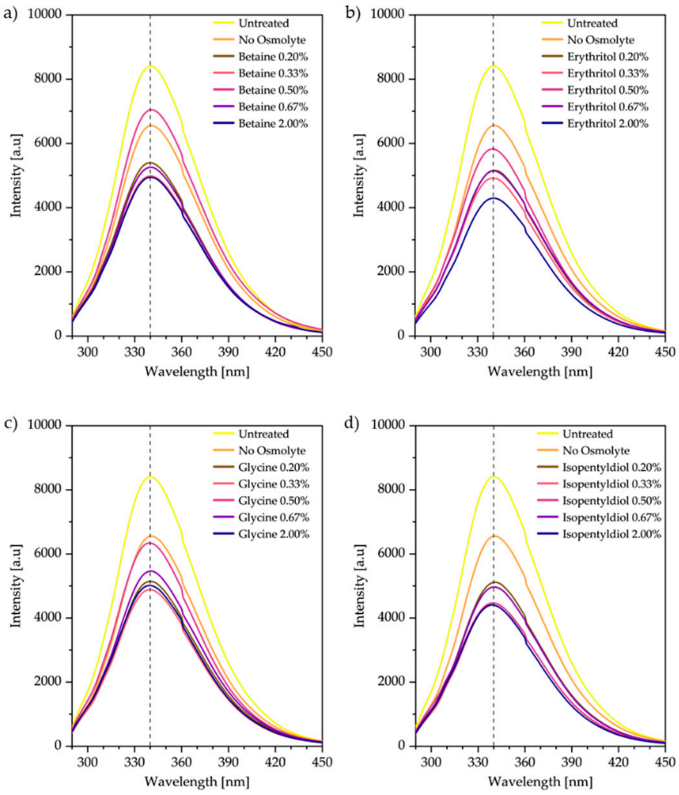

Figure S1. Evaluation of tertiary structure of BSA exploiting fluorescence emission in the presence or not of osmolyte at 0.20, 0.33, 0.50, 0.67 and 2.00% w/w by applying bleaching as stress effect: a) betaine, b) erythritol, c) glycine and d) isopentyldiol.

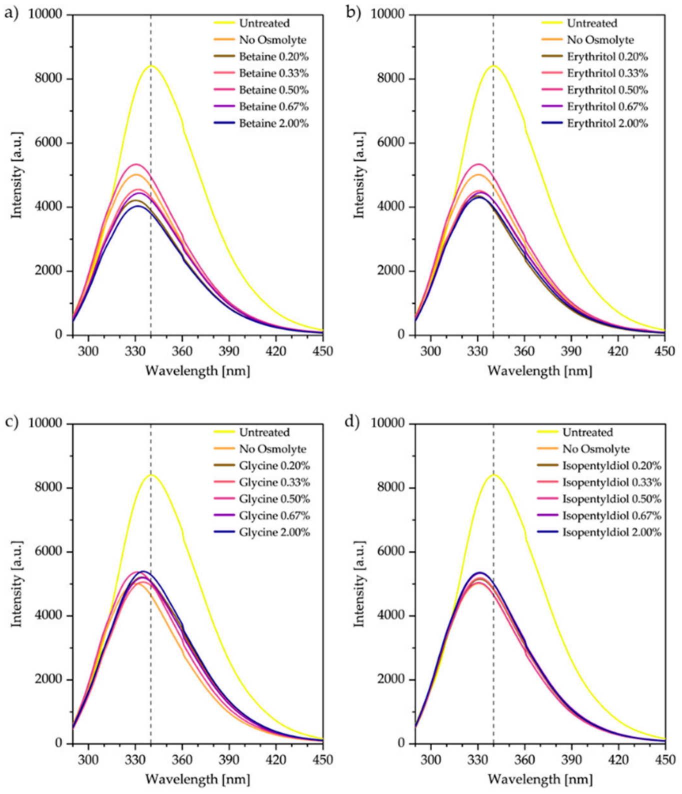

Figure S2. Evaluation of tertiary structure of BSA exploiting fluorescence emission in the presence or not of osmolyte at 0.20, 0.33, 0.50, 0.67 and 2.00% w/w by applying high-temperature (70 °C) as stress effect: a) betaine, b) erythritol, c) glycine and d) isopentyldiol.

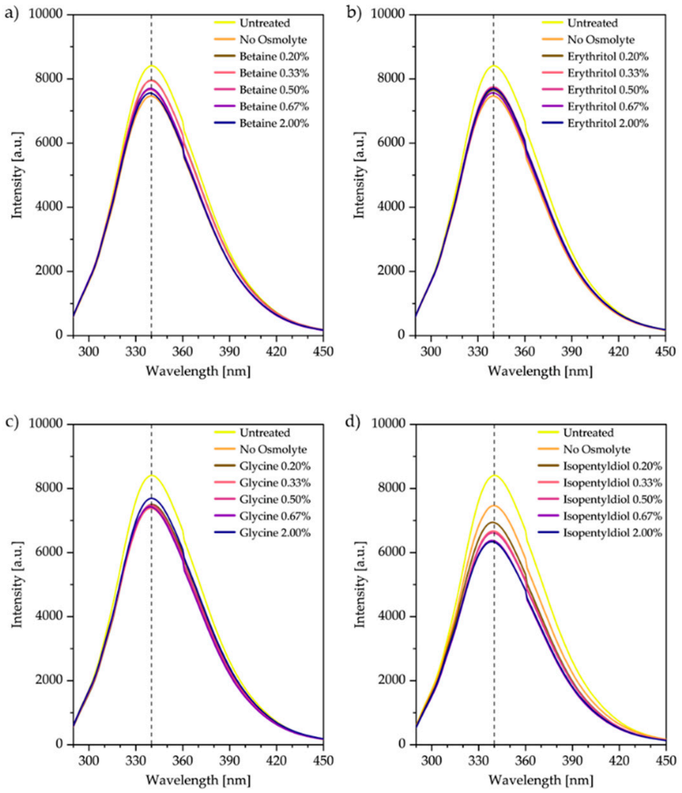

Figure S3. Evaluation of tertiary structure of BSA exploiting fluorescence emission in the presence or not of osmolyte at 0.20, 0.33, 0.50, 0.67 and 2.00% w/w by applying low-temperature (4 °C) as stress effect: a) betaine, b) erythritol, c) glycine and d) isopentyldiol.

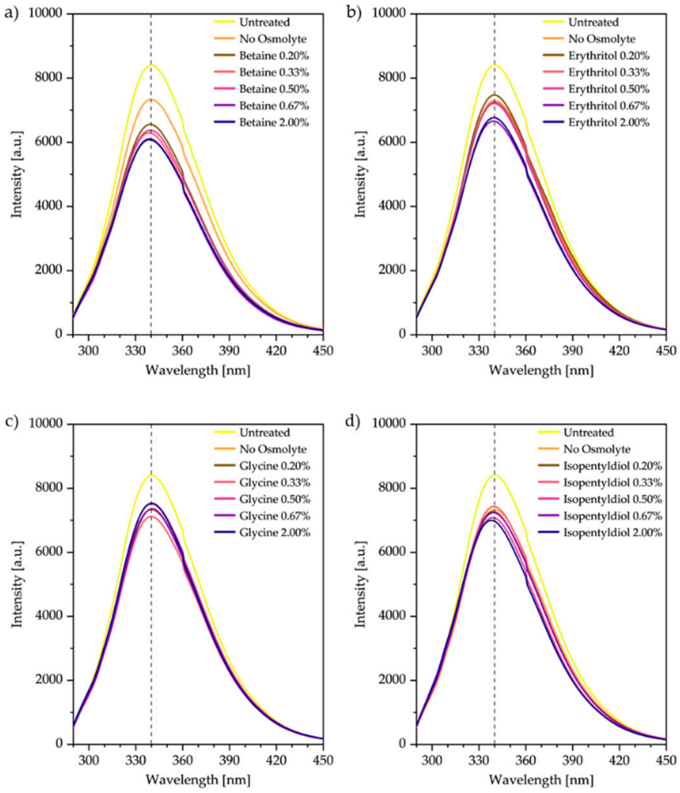

Figure S4. Evaluation of tertiary structure of BSA exploiting fluorescence emission in the presence or not of osmolyte at 0.20, 0.33, 0.50, 0.67 and 2.00% w/w by applying UVA radiation as stress effect: a) betaine, b) erythritol, c) glycine and d) isopentyldiol.

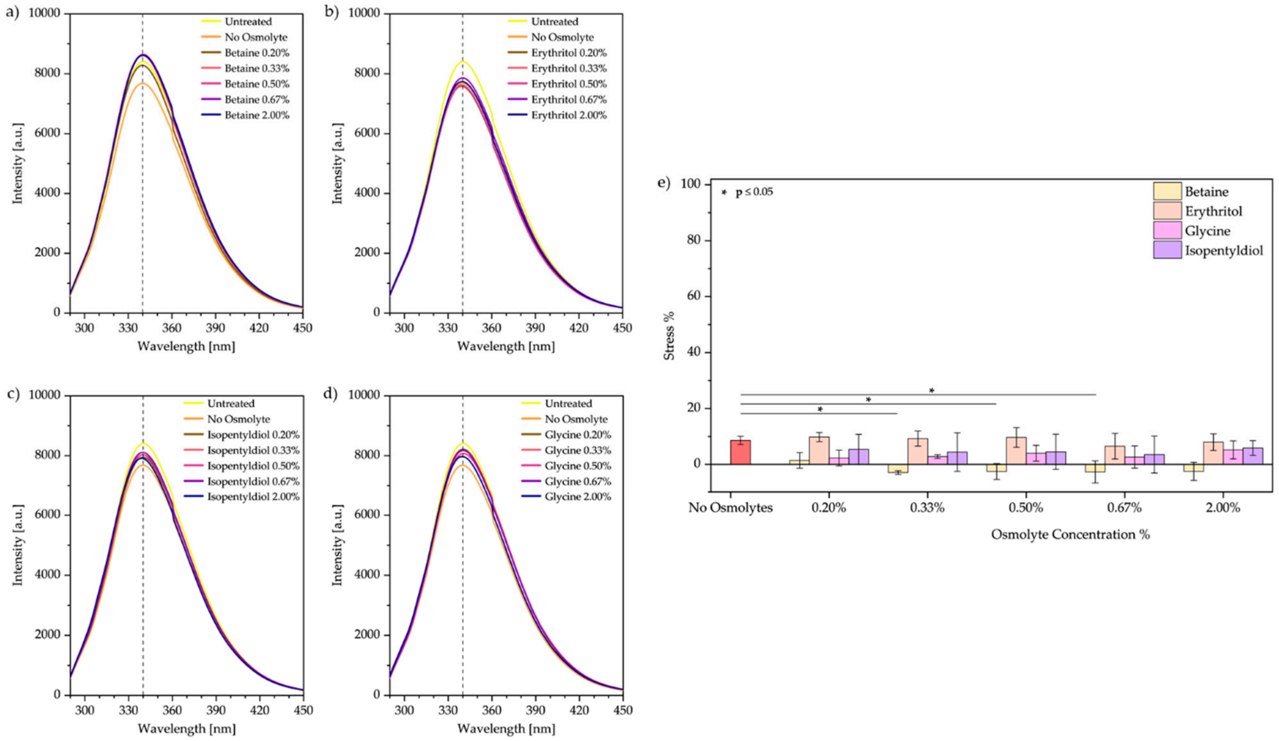

Figure S5. Evaluation of tertiary structure of BSA exploiting fluorescence emission in the presence or not of osmolyte at 0.20, 0.33, 0.50, 0.67 and 2.00% w/w by applying UVC radiation as stress effect: a) betaine, b) erythritol, c) glycine and d) isopentyldiol. e) Stress percentage comparison of BSA in the presence or not of osmolyte at 0.20, 0.33, 0.50, 0.67 and 2.00% w/w. Data represent mean ± SD (n = 3). P-value, *$p < 0.05$, compared to the control (No Osmolytes), as indicated by horizontal lines with asterisks. The absence of a line indicates no significant difference.

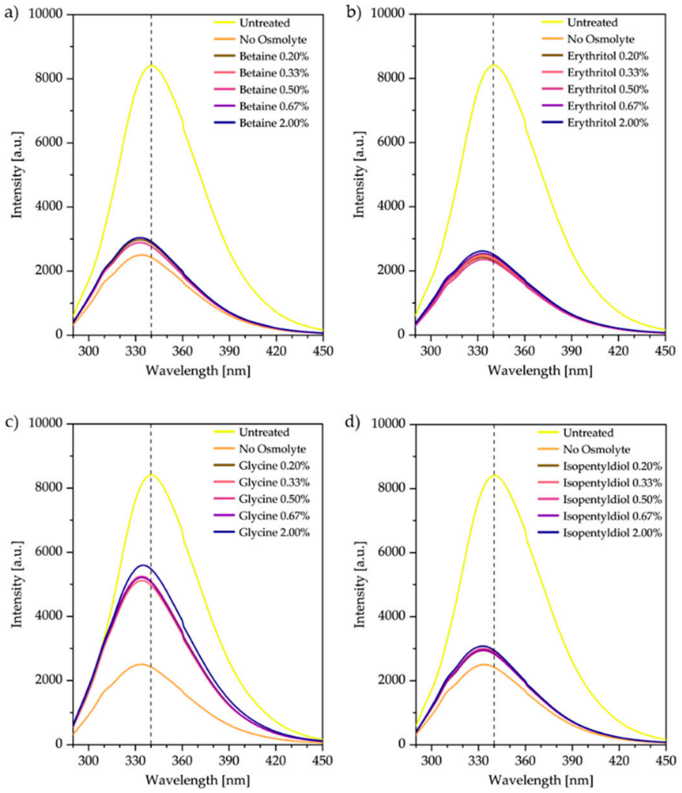

Figure S6. Evaluation of tertiary structure of BSA exploiting fluorescence emission in the presence or not of osmolyte at 0.20, 0.33, 0.50, 0.67 and 2.00% w/w by applying basic solution (pH 12) as stress effect: a) betaine, b) erythritol, c) glycine and d) isopentyldiol.

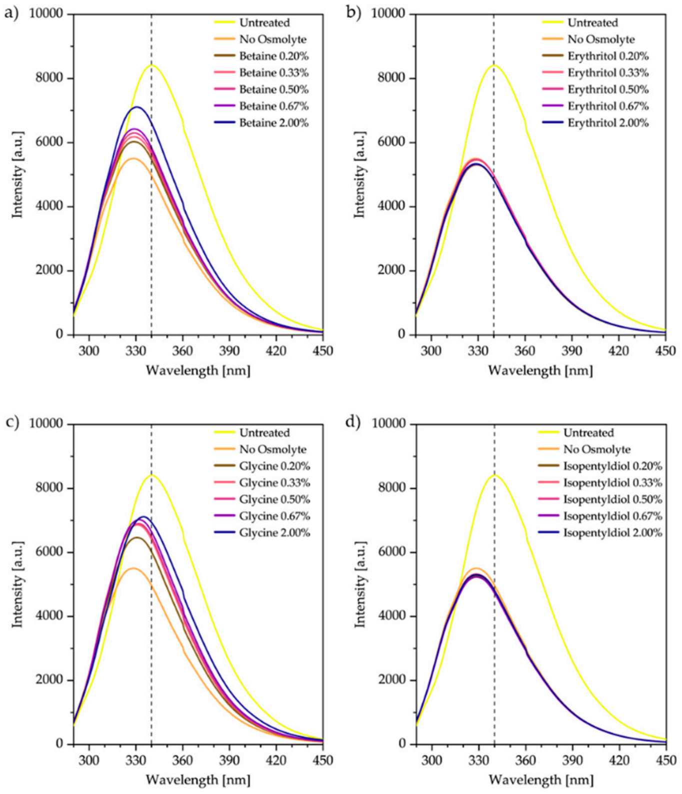

Figure S7. Evaluation of tertiary structure of BSA exploiting fluorescence emission in the presence or not of osmolyte at 0.20, 0.33, 0.50, 0.67 and 2.00% w/w by applying acid solution (pH 2) as stress effect: a) betaine, b) erythritol, c) glycine and d) isopentyldiol.

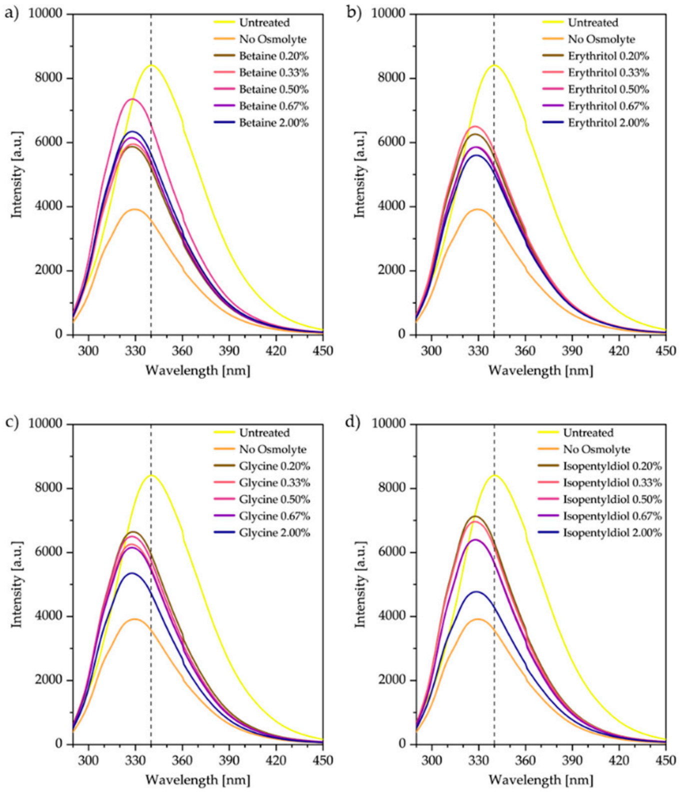

Figure S8. Evaluation of tertiary structure of BSA exploiting fluorescence emission in the presence or not of osmolyte at 0.20, 0.33, 0.50, 0.67 and 2.00% w/w by applying acid peeling solution as stress effect at the same time: a) betaine, b) erythritol, c) glycine and d) isopentyldiol.

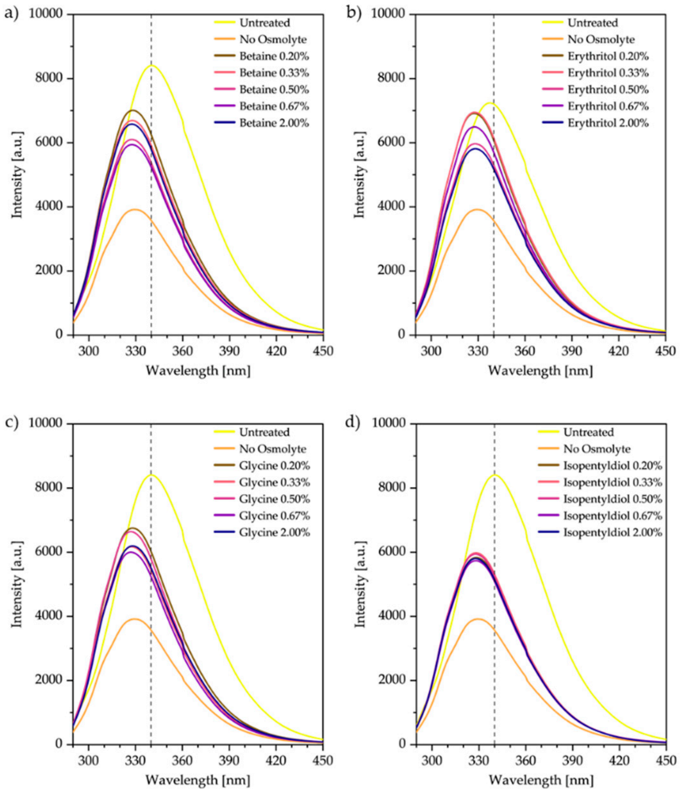

Figure S9. Evaluation of tertiary structure of BSA exploiting fluorescence emission by applying acid peeling solution as stress effect after treatment of osmolyte at 0.20, 0.33, 0.50, 0.67 and 2.00% w/w: a) betaine, b) erythritol, c) glycine and d) isopentyldiol.

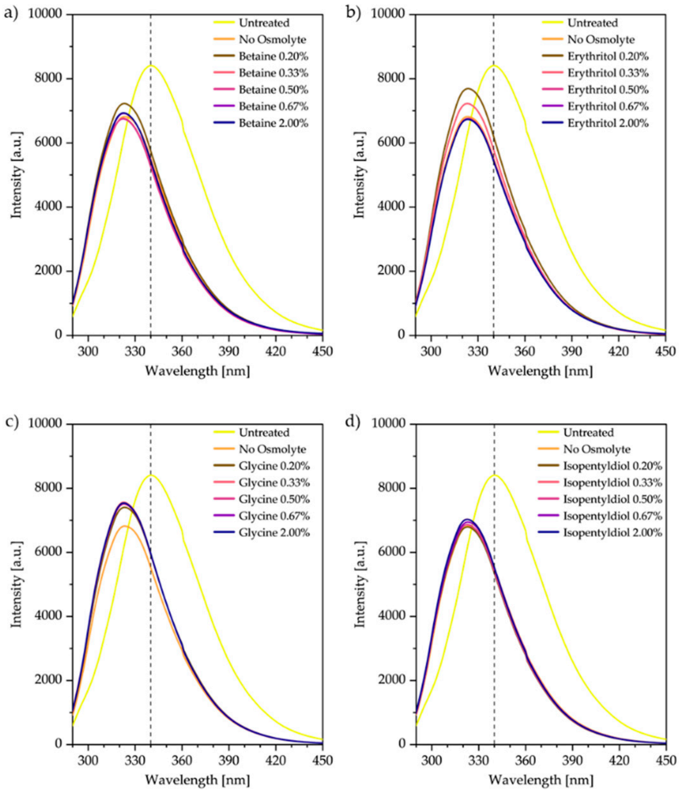

Figure S10. Evaluation of tertiary structure of BSA exploiting fluorescence emission in the presence or not of osmolyte at 0.20, 0.33, 0.50, 0.67 and 2.00% w/w by applying SLES solution as stress effect: a) betaine, b) erythritol, c) glycine and d) isopentyldiol.

Fluorescence emission of osmolytes at the maximum concentration (2.00% w/w) was acquired to exclude interference with signals obtained in the previous tests.

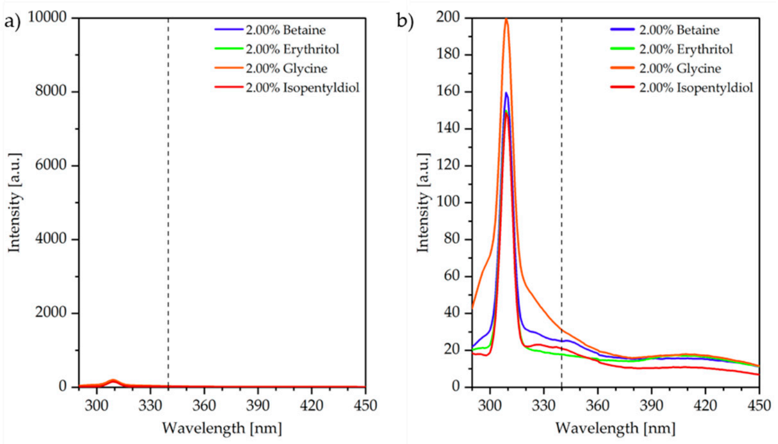

Figure S11. Fluorescence emission of osmolytes at 2.00% w/w. a) Maximum intensity value (Y-Axis) of 10000; b) Maximum intensity value (Y-Axis) of 200.

2. UV-Vis analysis on BSA under stress conditions

UV-Vis spectroscopy was employed to compare the BSA spectrum after each stress condition applied in the full study. Although UV spectroscopy is not the primary technique for analyzing secondary structure and denaturation, variations in absorbance can provide indirect information about conformational changes. The loss of secondary or tertiary structure can influence absorbance, especially when exposed to solvents, heat, or pH variations. The blank was the corresponding solution treated under the specific condition. We observed that oxidation played a significant role in the protein, leading to the disappearance of the peak at 280 nm. Fluorescence emission measurements revealed approximately 20% stress, which the higher sensitivity of the technique can explain. In all other cases, the shape and intensity of the absorption increased, which can be attributed to a different environment caused by changes in pH and temperature. These processes might expose aromatic groups (such as tryptophan, tyrosine, and phenylalanine) previously hidden within the protein's hydrophobic core. The increased exposure of these groups leads to greater UV absorption at 280 nm. Denatured proteins tend to form aggregates through hydrophobic interactions and disulfide bonds. These aggregates can alter the UV absorption profile, leading to an apparent increase in absorbance. When the protein forms large aggregates, an increase in absorbance due to light scattering can be observed. This phenomenon is not a true increase in UV absorption but contributes to the signal.

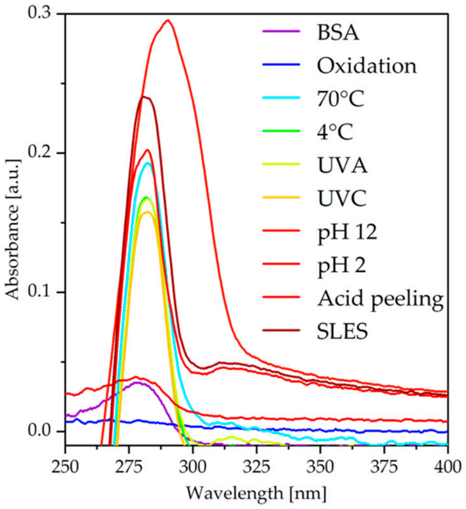

Figure S12. Absorbance of BSA under stress conditions noticing different behaviour at each condition.

3. FT-ATR analysis on BSA under stress conditions

Bovine Serum Albumin (BSA) was analyzed using Fourier Transform Attenuated Total Reflectance (FT-ATR) spectroscopy, revealing three characteristic transmission bands: the Amide I band (approximately 1650 cm^{-1}), associated with carbonyl stretching vibrations; the Amide II band (near 1550 cm^{-1}), attributed to a combination of C–N stretching and N–H bending in secondary amides; and a C–H bending band at 1450 cm^{-1}. Following each treatment, BSA was purified to remove residual molecules, lyophilized, and subjected to IR spectroscopic analysis. To study the effects of various stress conditions, samples were designated as follows: (b) oxidation, (c) 70°C, (d) 4°C, (e) UVA exposure, (f) UVC exposure, (g) alkaline pH (pH 12), (h) acidic pH (pH 2), (i) acid peeling, and (j) treatment with SLES. The results are summarized in Figure 13S, which compares the spectrum of not-treated BSA (a) to those subjected to different stress conditions (b–j). The analysis revealed that the stress conditions significantly affected the BSA structure. Notably, only the sample treated under acidic conditions (pH 2) exhibited a spectrum comparable to untreated BSA. In samples b, c, d, e, f, and i, additional peaks emerged between 1250 and 750 cm^{-1}, attributed to aromatic amino acids being exposed to a more hydrophobic environment. UVA and UVC treatments also resulted in the appearance of these peaks. Under oxidative conditions (b), an increase in the intensity of the band at 3250 cm^{-1} was observed, potentially indicating increased exposure of $CONH_2$ groups. Data acquired by Bruker INVENIO® S FT-IR.

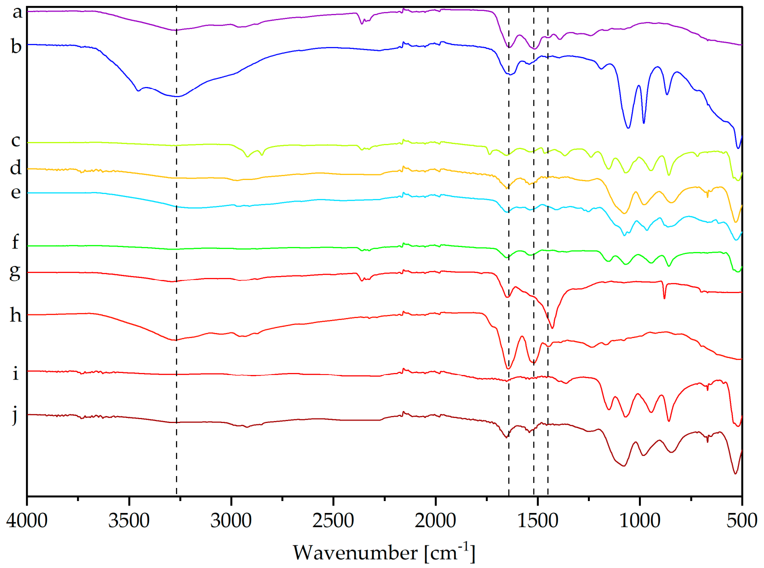

Figure S13. FT-ATR of BSA under stress conditions: (a) BSA not treated, (b) oxidation, (c) 70°C, (d) 4°C, (e) UVA, (f) UVC, (g) alkaline pH (pH 12), (h) acidic pH (pH 2), (i) acid peeling, and (j) SLES.

4. FT-ATR analysis on Erythritol after oxidation treatment

The amount of erythritol used in some tests (2% w/w concentration) was analyzed in the presence of H_2O_2 under the same conditions as the experiment. Following the reaction, the product was lyophilized and analyzed using IR spectroscopy. A comparison of the spectra obtained before (erythritol) and after the oxidative treatment revealed differences, indicating that erythritol participates in a redox reaction, forming erythrose as a byproduct during the oxidative test in the presence of BSA. A change in the spectrum was observed, particularly at 1672 cm^{-1}, attributed to the C=O stretching of the aldehyde group. Data acquired by Bruker INVENIO® S FT-IR.

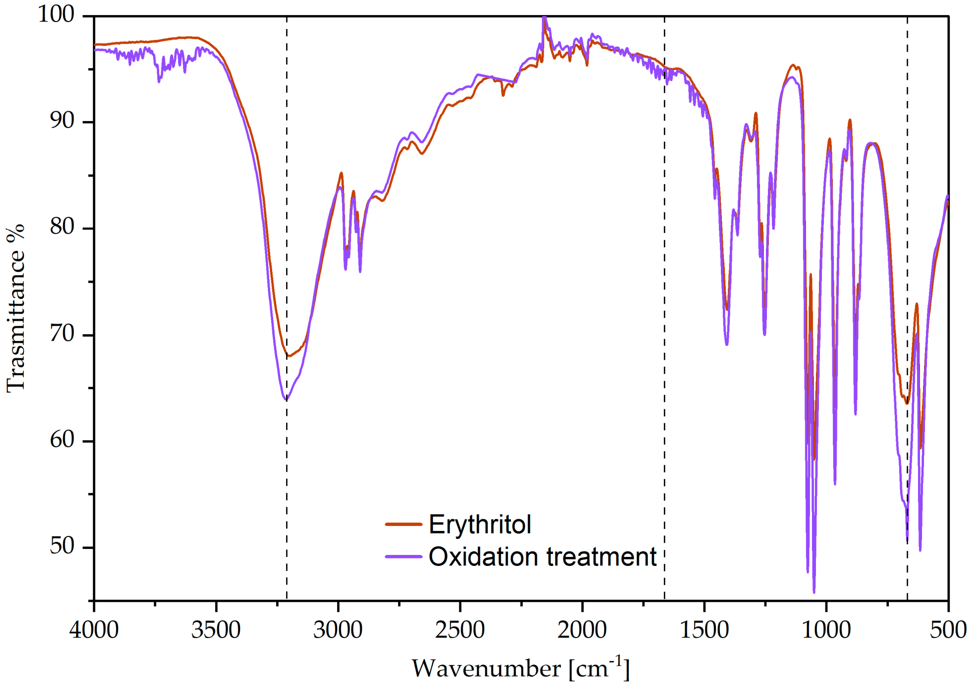

Figure S14. FT-ATR of Erythritol before and after oxidation treatment.
